# Development of a Novel Debittering Process for *Xanthoceras sorbifolium* Kernels to Preserve High Nutrition and Superior Flavor

**DOI:** 10.1002/fsn3.71807

**Published:** 2026-04-20

**Authors:** Yan Chen, Yi Fang, Xiaowei Zhang, Xin Wang, Yong Wu, Shuangyan Zheng, Jinyan Gao, Hongbing Chen

**Affiliations:** ^1^ State Key Laboratory of Food Science and Resources Nanchang University Nanchang China; ^2^ College of Food Science and Technology, Nanchang University Nanchang China; ^3^ Sino German Joint Research Institute Nanchang University Nanchang China; ^4^ International Institute of Food Innovation Nanchang University Nanchang China

**Keywords:** debittering technology, macronutrient preservation, synergistic extraction, *Xanthoceras sorbifolium* kernels

## Abstract

The high‐value utilization of *Xanthoceras sorbifolium (X. sorbifolium) kernels* is currently constrained by the presence of intense bitter compounds, primarily trigonelline. This study presents a novel, solvent‐based debittering process designed to achieve a synergistic balance between complete bitterness removal and the preservation of structural and nutritional integrity. Through systematic optimization of ethanol concentration, extraction time, and temperature, the process achieved a remarkable 93.5% reduction in trigonelline content (from 314.0 to 20.5 mg/kg), resulting in a bitterness score of zero. Crucially, the debittered kernels maintained their structural integrity with exceptional retention of key nutrients, including proteins (98.90%) and lipids (95.91%). Comparative analysis confirmed that the optimized process effectively eliminates bitterness while preserving the kernels' desirable sensory attributes and nutritional profile. These findings provide a robust and practical technical framework for the industrial‐scale processing and value‐added application of *X. sorbifolium* kernels in the food industry.

## Graphical abstract

Develop a novel debittering process for *Xanthoceras sorbifolium* kernels with retained high nutrition and superior flavor.

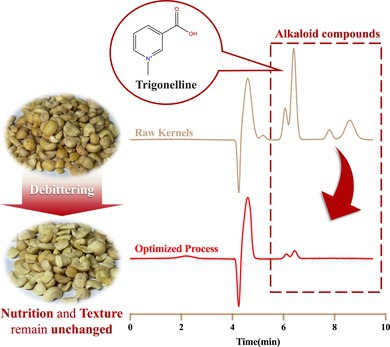

## 1 Introduction

*Xanthoceras sorbifolium* (*X. sorbifolium*) is a woody oil crop unique to China, whose kernels are highly valued for their nutritional composition, approximately 26% protein, 56% fat, 18% dietary fiber, as well as 16 essential amino acids, 15 trace elements, and 5 vitamins (Cui et al. 2024). Therefore, it serves as a high‐quality supplementary source of nut fatty acids (Chen and Zhang 2017). Since the kernel of the *X. sorbifolium* was officially approved as a new food raw material by China's National Health Commission in 2023, it has shown great potential for market development (Chen and Zhang 2017). However, its commercial application has been substantially constrained by complex bitter compounds (alkaloids, phenolics, and terpenoids) accumulated during late maturation, with industry surveys indicating less than 30% market conversion rate for terminal products due to bitterness issues (Pang et al. 2023).

Research reveals that the bitterness originates from synergistic effects of multiple secondary metabolites: (1) Alkaloids (e.g., Torreyanine) induce persistent bitterness through activation of T2R bitter taste receptors; (2). Phenolic compounds (flavonoids and tannins) not only impart astringent bitterness but also enhance taste perception through salivary protein binding; (3) Specific amino acids (glutamic acid and arginine) generate bitter derivatives via Maillard reaction during thermal processing (Kok et al. 2018; Chen et al. 2020). Current debittering techniques predominantly employ solvent extraction (methanol/ethanol extraction efficiency: 65%–78%) (Evangelou et al. 2018), but suffer from excessive nutrient loss (15%–20% protein depletion) and kernel structural damage (Tan and Seetoh 2022).

This study focuses on the debittering technology of ready‐to‐eat *X. sorbifolium* kernels. At present, no such commercial products are available, which require intact kernels, favorable sensory properties, and high macronutrient (protein and fat) preservation. However, existing processing mainly targets oil extraction and sacrifices nutrients to improve oil yield (Ruan et al. 2017; Gao et al. 2024). Conventional thermal processing only reduces bitterness by 15%–20% but lowers vitamin B_1_ retention to 63.5% (Zhou et al. 2023; Tambo Téné et al. 2025). The intrinsic bitterness severely limits the industrial application of this nutrient‐dense resource. This study first identified trigonelline as the primary contributor to the bitter taste of the kernels. Building on this finding, an innovative debittering process was developed, which achieves nearly complete elimination of bitterness while concurrently preserving kernel structural integrity, maintaining high contents of proteins and fats, and ensuring favorable flavor profiles. The proposed method addresses the key challenge of balancing debittering efficiency with nutrient retention, providing a promising technical solution for the value‐added utilization of *X. sorbifolium* kernels.

## 2 Materials and Methods

### 2.1 Materials

Sodium citrate (Sinopharm Chemical Reagent Co. Ltd.); petroleum ether (Sinopharm Chemical Reagent Co. Ltd., analytically pure); fenugreek base (Sigma Chemical Co., St. Louis, MO, USA); sodium citrate (Sinopharm Chemical Reagent Co. Ltd.).

### 2.2 Identification of Bitter Components

For catechin determination: Ground *X. sorbifolium* seeds (0.2 g, ±0.0001 g) were twice extracted with 5 mL preheated 70% methanol (70°C) via 10‐min water bath extraction (stirred every 5 min); after centrifugation (3500 r/min, 10 min), combined supernatants were made up to 10 mL, filtered through a 0.45 μm organic membrane, and a 2 mL aliquot was diluted to 10 mL with a stabilizing solution (EDTA‐2Na + ascorbic acid + acetonitrile + water) before re‐filtration. HPLC analysis used a C18 column (5 μm, 250 mm × 4.6 mm) at 35°C, with 1 mL/min flow rate, 278 nm detection wavelength, 10 μL injection volume, and gradient elution (0–10 min: 100% phase A; 10–25 min: 100% → 68% A; 25–35 min: 68% A, followed by re‐equilibration). For trigonelline determination: Ground seeds (1–2 g, ±0.001 g) were extracted with 40 mL boiling water for 30 min; after filtration, the filtrate was partitioned with 20 mL n‐hexane, the aqueous phase was transferred to a 50 mL volumetric flask, mixed with 0.5 mL 50 g/L sulfosalicylic acid solution, made up to volume, and the supernatant was filtered through a 0.45 μm aqueous membrane. Its HPLC analysis used an amino column (5 μm, 250 mm × 4.6 mm) at 30°C, with 0.80 mL/min flow rate, 260 nm detection wavelength, 10 μL injection volume, and isocratic elution with methanol–water (88:12, v/v). Both components used calibration curves (catechins: mixed standards of +C, EC, EGC, EGCG, ECG at specified concentrations; trigonelline: 1.0–50.0 mg/L standards) for quantification via calibration curve or single‐point calibration (with reagent blank control), and content calculation by: $X=\frac{C\times V}{m\times 10000}$ (X: g/100 g; C: μg/mL; V: mL; m: g), relative deviation ≤ 10% (two decimal places) for catechins and absolute difference ≤ 15% of the mean (three significant figures) for trigonelline.

### 2.3 Debittering Process

In this study, 200 g of undamaged fresh *X. sorbifolium* kernels were selected and mixed with 400 g of water for gradient debittering. *X. sorbifolium* kernels were harvested from the Arshan Mountains in Ulanhot, Inner Mongolia.

The pretreated samples were subsequently mixed with a specific ratio of organic solvents for extraction. The experimental group used an ethanol‐water system as the extraction solvent. Preliminary screening indicated that ethanol offered the best balance of debittering efficiency and safety. Therefore, the optimization experiments focused on ethanol concentration (10%–60%), solid–liquid ratio (1:2–1:5), extraction time (4–20 h), and temperature (0°C–60°C). Finally, all samples were rinsed with water and uniformly dried at 100°C in hot air to complete the debittering process.

### 2.4 Electronic Tongue Sensor Array

For sample pretreatment, 1.00 g of *X. sorbifolium* seed kernel powder (three parallels) was accurately weighed into beakers, each mixed with 100 mL ultrapure water, and stirred at 60°C for 30 min via magnetic stirrer to fully extract taste compounds. The extract was centrifuged at 8000 rpm for 10 min; the supernatant was collected, made up to 100 mL with ultrapure water, then its pH was measured and adjusted to 6.0 with dilute HCl/NaOH, homogenized with water at 1:10, centrifuged, filtered, and set for detection. For electronic tongue preparation, sensors followed the sequence: cleaning solution (90 s) → reference solution (120 s) → second reference solution (120 s) → equilibration/zeroing (30 s) → 30 s initial taste testing; after 3‐s re‐cleaning with reference solution, sensors tested aftertaste in new reference solution for 30 s. Bitter‐related sensors (C00, AE1, CA0, CT0, AAE, ANO, BTO) were tested four times (first cycle discarded, average of three later), and sweet sensor (GL1) five times (first/last discarded, average of three middle). For calibration and data acquisition, KCl reference and sample solutions were added to dedicated cups, with measurement sequence set as R1 → S → R2; sensors were immersed in R1 for baseline potential, S for response potential, and R2 for cleaning/stability check. Single measurement lasted 120 s (1 Hz acquisition), with Vs taken as the average potential of the most stable phase (e.g., last 10 s); each sample was measured ≥ 3 times randomly, and sensors cleaned with ultrapure water after each test. For data processing, the system calculated ΔV=Vs−Vr; bitter intensity was evaluated via *ΔV* comparison (higher *ΔV* = stronger taste), and PCA was used for multi‐sensor data dimensionality reduction, with PCA score plots showing taste profile differences and clustering of samples before/after debittering.

### 2.5 Nutrient Composition Test

The post‐soaking filter residue was subjected to extraction experiments under two distinct conditions. In the experimental groups, ultrasonic‐assisted extraction was carried out by using an ultrasonic cleaner (model KQ‐250 dB, power 200 W, frequency 40 kHz) produced by Shanghai Xinyu Instrument and Equipment Co. Ltd. The filter residue was placed in a cleaning bath containing 400 mL of distilled water (25°C), and the extraction time was 30 min. In the control groups, the extraction was performed by the conventional water bath heating method with static incubation at 45°C (same model water bath) for 30 min. Subsequently, the extract was filtered and the supernatant was collected using a high‐speed centrifuge (Shanghai Anting TDL‐5‐A, 4000 rpm, 10 min). Protein content was determined by Kjeldahl method using a digestion oven (Shanghai Jingke JDK‐405) and an automatic nitrogen analyzer (FOSS Kjeltec 2300). Fat content was determined by Soxhlet extraction using petroleum ether as solvent (Sinopharm Chemical Reagent Co. Ltd., analytically pure) in a Soxhlet extractor for 6 h of reflux extraction of dried samples. Changes in nutrient content and rate of loss were recorded at three stages: pre‐soaking, post‐soaking and post‐extraction.

### 2.6 Determination of Sensory Indicators

A texture analyzer was used for the systematic determination of the hardness, springiness, cohesiveness, gumminess, chewiness, and resilience of *X. sorbifolium* seed kernels, aiming to comprehensively evaluate their textural properties. For instrument calibration before testing: standard weights were used to calibrate the load, and standard modules were applied to calibrate the displacement; the instrument was put into use only after its accuracy met the testing requirements. For sample preparation, seed kernels from the same batch with uniform texture were selected as test samples, and their shape and size were required to be basically consistent to ensure the representativeness and accuracy of the test results. For probe selection: according to the morphological characteristics of *X. sorbifolium* seed kernels, the P/36R probe was chosen for the texture test. For parameter setting: combined with the tissue characteristics of *X. sorbifolium* seed kernels, the texture analyzer parameters were set as follows: preload of 10 g, test speed of 1.5 mm/s, return speed of 4 mm/s, compression depth of 10%, and data acquisition frequency of 200 pps. For the test process, the sample was placed in the center of the test platform, adjusted to align with the probe, and the equipment was started according to the preset parameters; the system automatically completed the test and recorded the corresponding data. Through this process, the texture analyzer systematically determined the hardness, springiness, cohesiveness, gumminess, chewiness, and resilience of *X. sorbifolium* seed kernels, so as to comprehensively assess their textural properties.

### 2.7 Statistical Analysis

Experiments were performed with specific replication numbers: *n* = 3 for single‐factor optimization, *n* = 7 for sensory evaluation, and *n* = 2 for general physicochemical analyses. Data are expressed as mean ± standard deviation (SD). Statistical analysis was performed using one‐way analysis of variance (ANOVA). Differences were considered statistically significant at *p* < 0.05. In the figures, significance levels are indicated by asterisks (*p* < 0.05, *p* < 0.01, *p* < 0.001, *p* < 0.0001), and ‘ns’ indicates no significant difference.

## 3 Results

### 3.1 Determination of Key Bitter Substances

**FIGURE 1:**
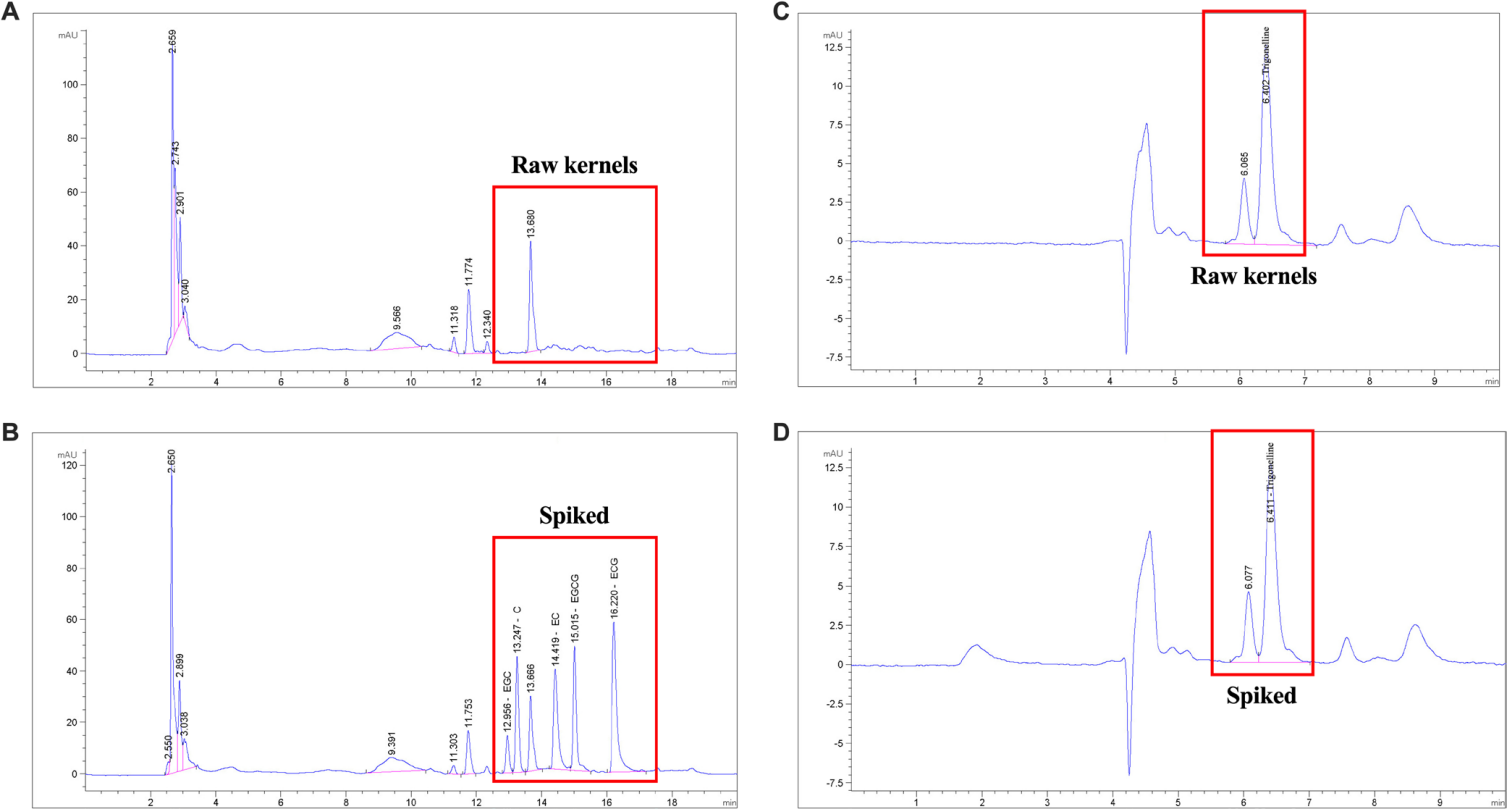
HPLC chromatograms for the identification of bitter compounds in *X. sorbifolium* kernels. (A) Chromatogram of catechins in the kernel of *X. sorbifolium*, before debittering. (B) Chromatogram spiked with catechin standards. (C) Chromatogram of alkaloid fraction in the kernel of *X. sorbifolium*, before debittering. (D) Chromatogram spiked with trigonelline standard.

To systematically investigate the key substances contributing to the bitter taste characteristic of *X. sorbifolium* kernels, high‐performance liquid chromatography (HPLC) was employed for quantitative analysis and spike recovery experiments targeting several major catechin compounds, including catechin (C), epicatechin (EC), epigallocatechin (EGC), epicatechin gallate (ECG), and epigallocatechin gallate (EGCG). The corresponding analytical results are presented in Figure 1A,B. Detection data revealed that the contents of the aforementioned catechin compounds in *X. sorbifolium* kernels were below the method's limit of detection (LOD), indicating that these substances are either absent in the actual samples or present at extremely low concentrations. Meanwhile, the spike recovery rate of the method for catechins was 105%, which verified the reliability of the analytical process. Based on these findings, it can be reasonably inferred that catechin components are not the primary contributors to the bitter taste of *X. sorbifolium* kernels. All relevant data supporting this conclusion are presented in Table 1.

**TABLE 1:** Quantification of bitter substances (epicatechins and trigonelline) in raw *X. sorbifolium* kernels via HPLC detection.

Project	*m* (g)	*C* (mg/L)	*V* (mL)	*X* (g/100 g)	Result (mg/kg)
Catechins					
Sample	5.032	0.00000	10	0.00000	0
Parallel samples	5.051	0.00000	10	0.00000	
Blank	0	0.00000	10	0.00000	0
Spiked recovery	5.118	11.61215	10	0.00453	Recovery 105%
Trigonelline					
Sample	1.408	9.22793	50	0.03277	314.3
Parallel samples	1.480	8.90540	50	0.03009	
Blank	0	0	50	0	0
Spiked recovery	1.529	13.61215	50	0.04451	Recovery 109%

*Note: X* represents the content of bitter substances in the sample; *C* represents the concentration of bitter substances in the sample; *V* represents the constant volume of the measured sample solution; *m* represents the weighed amount of the sample.

Subsequently, quantitative analysis of trigonelline in *X. sorbifolium* kernels was conducted via HPLC, and the results are illustrated in Figure 1C,D. The determined content of trigonelline was 314 mg/kg, with a spike recovery rate of 109%, confirming the accuracy of the quantitative method. Notably, this measured value is significantly higher than the trigonelline bitter taste perception threshold (27–69 mg/kg) reported in existing literature. Collectively, these results demonstrate that trigonelline is one of the key bioactive substances responsible for the bitter taste characteristic of *X. sorbifolium* kernels.

### 3.2 Various Process Optimizations for the Trigonelline Residue Content in the Kernels of *X. sorbifolium* Kernels

Given the favorable water solubility of trigonelline, aqueous‐based extraction alone was insufficient for effective trigonelline removal from *X. sorbifolium* kernels due to the raw material's high oil content, which hindered extraction while preserving kernel integrity. Ethanol was therefore selected as the extraction solvent for debittering, owing to its miscibility with water, trigonelline, and lipids.

**FIGURE 2:**
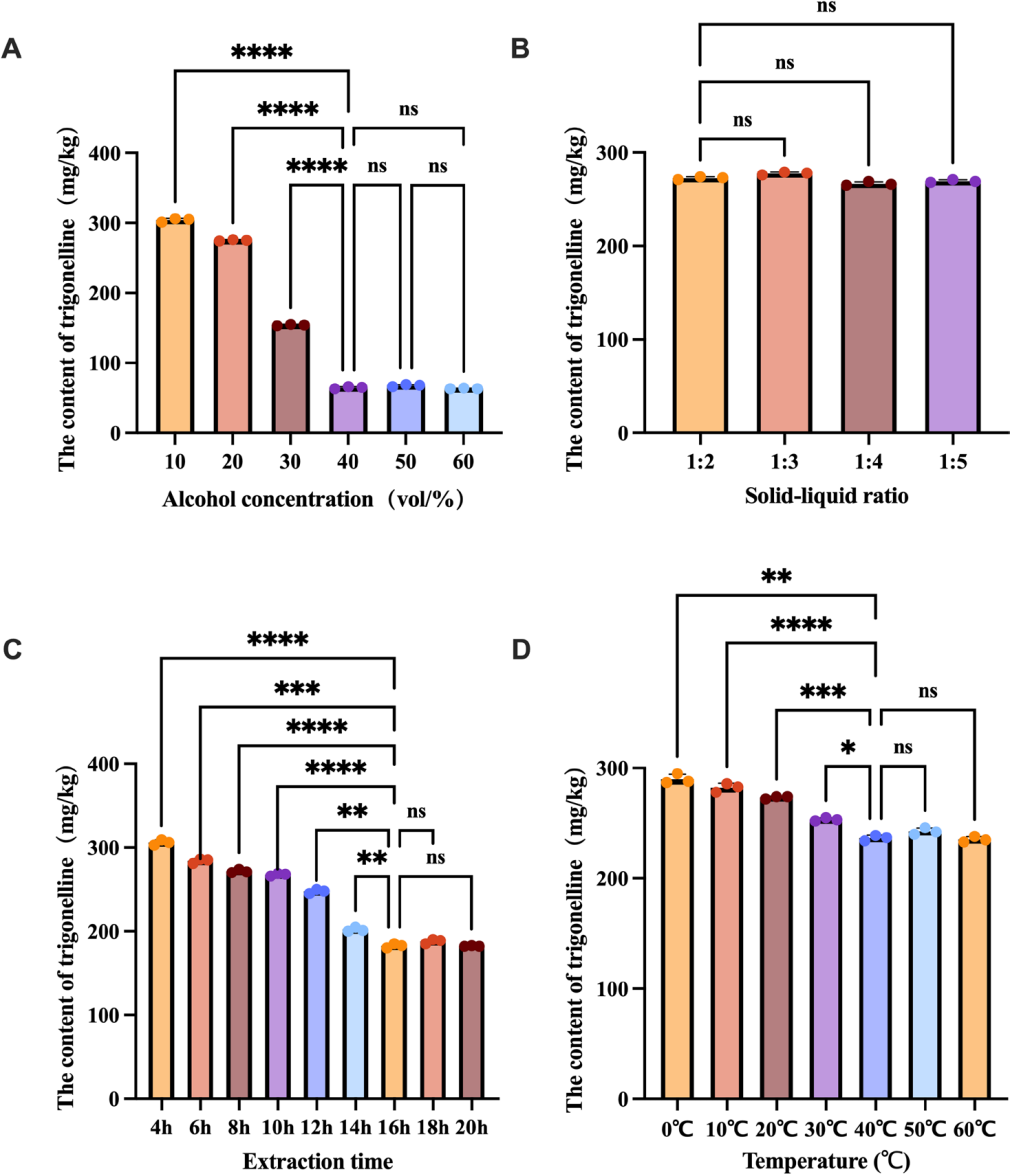
Optimization of the removal process of trigonelline from the kernels of *X. sorbifolium* kernels and analysis of its effect. (A) The effect of different ethanol concentrations on debittering effect (B) The effect of different material‐liquid ratios on debittering effect. (C) The effect of different extraction temperatures on debittering effect. (D) The effect of different extraction times on debittering effect. Data are expressed as mean ± SD (*n* = 3). *p < 0.05, **p < 0.01, ***p < 0.01, and ****p < 0.001.

Single‐factor experiments were conducted to investigate the effects of ethanol volume fraction (10%–60%, v/v), solid–liquid ratio, extraction temperature (0°C–60°C), and extraction time (2–24 h) on trigonelline removal efficiency, with results presented in Figure 2A–D. Under fixed solid–liquid ratio (1:2, w/v), extraction temperature (20°C), and extraction time (8 h), the trigonelline removal rate increased significantly with ethanol concentration from 10% to 40% but plateaued at concentrations exceeding 40% (Figure 2A). For the solid–liquid ratio (Figure 2B), removal efficiency reached a maximum at 1:2, with no significant improvements observed for further increases. Regarding extraction time (Figure 2C), under fixed solid–liquid ratio (1:2), ethanol concentration (20%, v/v), and extraction temperature (40°C), the removal rate increased significantly from 2 to 16 h and stabilized thereafter. Regarding the extraction temperature (Figure 2D), the optimal ratio determined in previous discussions was maintained—that is, the extraction temperature was investigated under the conditions of a fixed solid–liquid ratio of 1:2, ethanol volume fraction of 20%, and extraction time of 8 h. The removal rate increased significantly within the range of 0°C–40°C and remained stable above 40°C.

### 3.3 Orthogonal Experiments Yielded the Optimal Debittering Parameters

The optimal process parameters for trigonelline removal from *X. sorbifolium* kernels were determined via orthogonal experimental design based on an orthogonal array, as summarized in Table 2, which presents the results of the orthogonal experiment designed to minimize the residual trigonelline content. The Range analysis reveals the magnitude of each factor's effect: Ethanol Concentration (*R* = 11.91) > Extraction Time (*R* = 8.75) > Extraction Temperature (*R* = 2.13). The Range analysis value for ethanol confirms that solvent polarity is the most critical determinant for the solubility and removal of alkaloid‐type bitter compounds. Based on the mean effect values (*k*), the optimal conditions for minimizing residual trigonelline (i.e., achieving the lowest *k* value) were identified as 60% ethanol (*k*_*3*_ = 27.63), 20 h extraction time (*k*_*3*_ = 28.85), and 30°C temperature (*k*_*1*_ = 34.1).

**TABLE 2:** Orthogonal experimental design (L_9_(3^3^)) and range analysis for optimizing the removal of trigonelline from *X. sorbifolium* kernels.

Sample	A ethanol concentration	B extraction time	C extraction temperature	Residual amount of trigonelline mg/kg
1	50	16	50	45.2
2	40	18	50	41.8
3	50	18	40	38.5
4	50	18	40	42.3
5	50	18	40	36.7
6	40	18	30	33.9
7	40	20	40	31.2
8	60	18	30	28.6
9	40	16	40	35.4
10	50	20	30	34.8
11	60	16	40	30.5
12	50	16	30	39.1
13	50	18	40	37.4
14	60	20	40	23.5
15	50	18	40	36.2
16	60	18	50	29.8
17	50	20	50	32.6
k1	35.58	37.6	34.1	—
k2	37.34	35.73	35.22	—
k3	27.63	28.85	36.23	—
R	11.91	8.75	2.13	—

**FIGURE 3:**
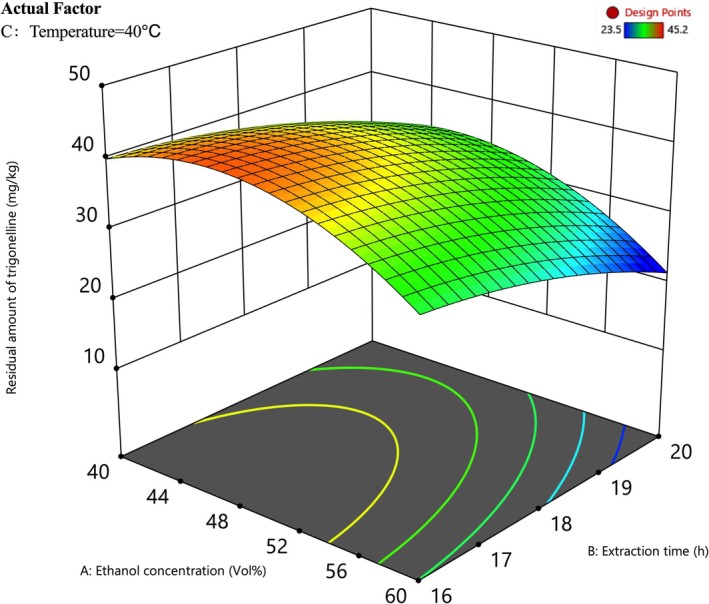
Response surface 3D plot and corresponding contour plot for the interactive effect of ethanol concentration and extraction time on the residual trigonelline content.

The response surface plot (Figure 3) further visualizes the interaction between the two dominant factors: ethanol concentration and extraction time. The 3D surface exhibits a steep downward slope as ethanol concentration increases from 40% to 60%, indicating a significant reduction in residual trigonelline. Similarly, extending the extraction time to 20 h facilitates better removal, likely due to establishing a more complete mass transfer equilibrium. In contrast, temperature showed a relatively flat response profile (*R* = 2.13), suggesting that the debittering process is less sensitive to thermal variations within the tested range (30°C–50°C). This low thermal sensitivity is advantageous for industrial applications, allowing the process to be conducted at lower temperatures to preserve heat‐sensitive nutrients and reduce energy consumption.

**FIGURE 4:**
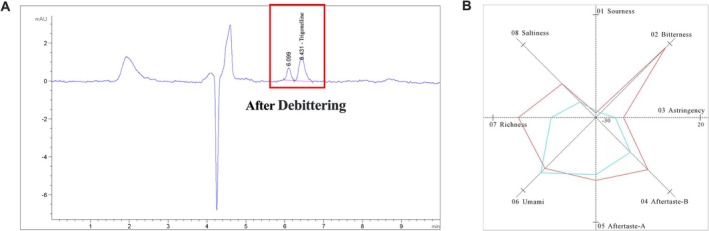
Changes in trigonelline after debittering and corresponding changes in flavor values. (A) The content change of trigonelline after debittering was verified and compared using HPLC. (B) Detection results of bitterness values of *X. sorbifolium* kernels before and after debittering (red indicates before debittering, blue indicates after debittering).

Quantitative analysis of trigonelline content before and after debittering was performed using HPLC, with the results presented in Figure 4A and Table 3. The initial trigonelline content in raw *X. sorbifolium* kernels was 314 mg/kg, which significantly decreased to 20.5 mg/kg after treatment under the optimized conditions. Correspondingly, the trigonelline removal efficiency reached 93.49%, demonstrating a remarkable debittering effect that effectively mitigates the bitter taste characteristic of *X. sorbifolium* kernels.

**TABLE 3:** Residual content of trigonelline in *Xanthoceras sorbifolium* kernels after the optimized debittering process.

Project	*m* (g)	*C* (mg/L)	*V* (mL)	*X* (g/100 g)	Result (mg/kg)
Sample	1.753	0.76834	50	0.00219	20.5
Parallel samples	1.487	0.56892	50	0.00191	

*Note: X* represents the content of bitter substances in the sample; *C* represents the concentration of bitter substances in the sample; *V* represents the constant volume of the measured sample solution; *m* represents the weighed amount of the sample.

### 3.4 Flavor Changes in Bitterness Value Before and After Debittering Treatment

As illustrated in Figure 4B, under the optimal debittering conditions (ethanol concentration: 60 vol%, extraction time: 20 h, extraction temperature: 40°C), electronic tongue technology was employed to quantitatively determine the bitterness value of *X. sorbifolium* kernel samples before and after treatment. The bitterness value of the samples decreased significantly from 5 (pre‐treatment) to 0 (post‐treatment), achieving significant reduction (> 93% removal of trigonelline).

On the radar chart's bitterness axis (02 Bitterness), the pre‐debittered sample (red curve) showed a significantly higher value (data point near the outer ring, scale: −30 center to 20 outer ring), indicating intense bitterness. In contrast, the post‐debittered sample (blue curve) exhibited a marked bitterness reduction (data point shifted toward the center), with a reduction rate exceeding 70% (e.g., 15–20 pre‐treatment vs. 0–5 post‐treatment). Astringency (03 Astringency) decreased synchronously with bitterness, while umami (06 Umami) and richness (07 Richness) remained stable or slightly increased (blue curve extended outward on corresponding axes), confirming the debittering process eliminated bitterness without compromising desirable flavor components.

These results validate the optimized process's superiority, providing critical technical support for the in‐depth development and food application of *X. sorbifolium* kernels. Complete bitterness removal enhances sensory acceptability and broadens their food industry prospects.

### 3.5 Changes in Nutrient Content of *X. sorbifolium* Kernels Before and After Debittering

As presented in Table 4, a comparative analysis of three major macronutrients (protein, fat, and carbohydrates) was conducted on *X. sorbifolium* kernels before and after debittering under the optimized process conditions. The results revealed that the total macronutrient content per 100 g of kernels decreased by 2.9 g post‐treatment, with specific reductions of 2.5 g in fat, 0.3 g in protein, and 0.1 g in carbohydrates.

**TABLE 4:** Comparison of nutritional components (fat, protein, and carbohydrates) in *X. sorbifolium* kernels before and after the debittering treatment.

Project	Before debittering	After debittering	Retention rate
Fat/g	61.1	58.6	95.91%
Protein/g	27.2	26.9	98.90%
Carbohydrates/g	9.0	8.9	98.89%

Overall, the debittering treatment exerted a limited impact on macronutrient retention and did not cause a significant diminishment of the overall nutritional value of *X. sorbifolium* kernels. This finding underscores the feasibility of the optimized debittering process for preserving the nutritional integrity of the raw material while achieving effective bitterness removal.

### 3.6 Changes in Sensory Quality of *X. sorbifolium* Kernels Before and After Debittering

After debittering *X. sorbifolium* kernels under optimized conditions, texture profile analysis and sensory evaluation were conducted to assess textural properties and sensory attributes (Figure 6).

**FIGURE 5:**
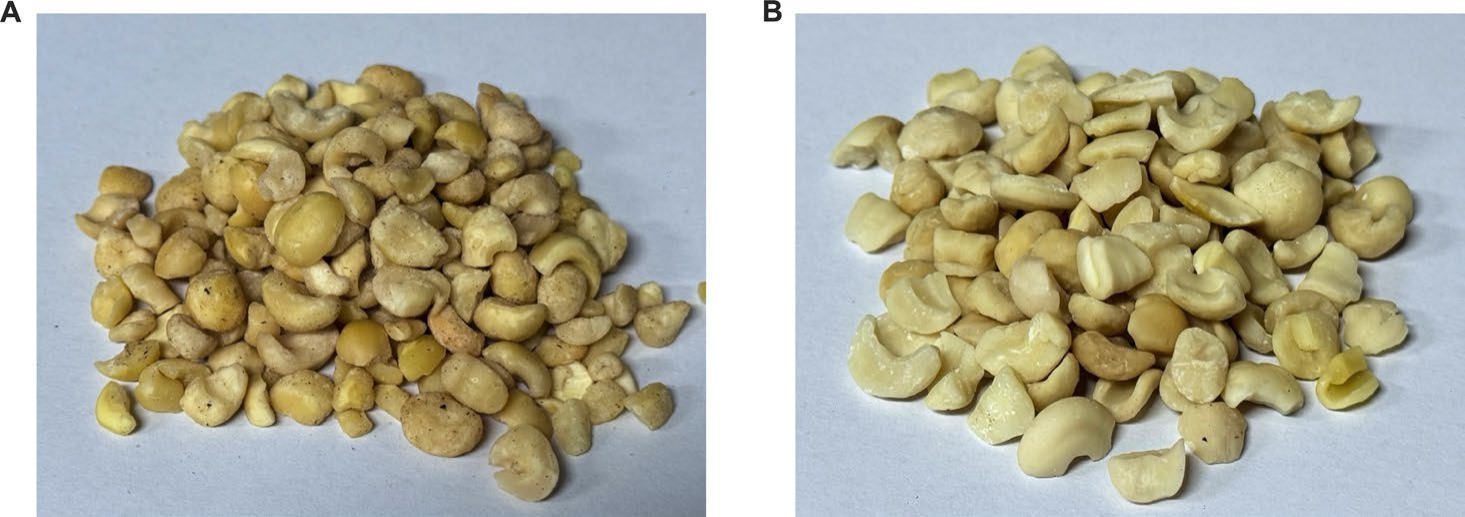
Comparison of surface texture of *X. sorbifolium* kernels before and after debittering. (A) Before debittering of *X. sorbifolium* kernels; (B) After debittering of *X. sorbifolium* kernels.

As shown in Figure 5, a comprehensive observation and comparative inspection was conducted on the surface color, odor, texture, and form of the kernels from four aspects to evaluate the changes in the texture characteristics of the nut surface. The inspection results are shown in Table 5. After treatment, the kernels maintained a relatively smooth and intact surface structure, comparable to that of the control group. The color remained milky white to ivory; the odor was the aroma of roasted almonds and light milk; the texture was initially brittle, becoming soft and tender after chewing; and the form was a plump and complete shape, with a long diameter of 8–12 mm and obvious longitudinal stripes on the surface. This indicates that the optimized debittering process did not cause significant physical damage to the kernel matrix.

**FIGURE 6:**
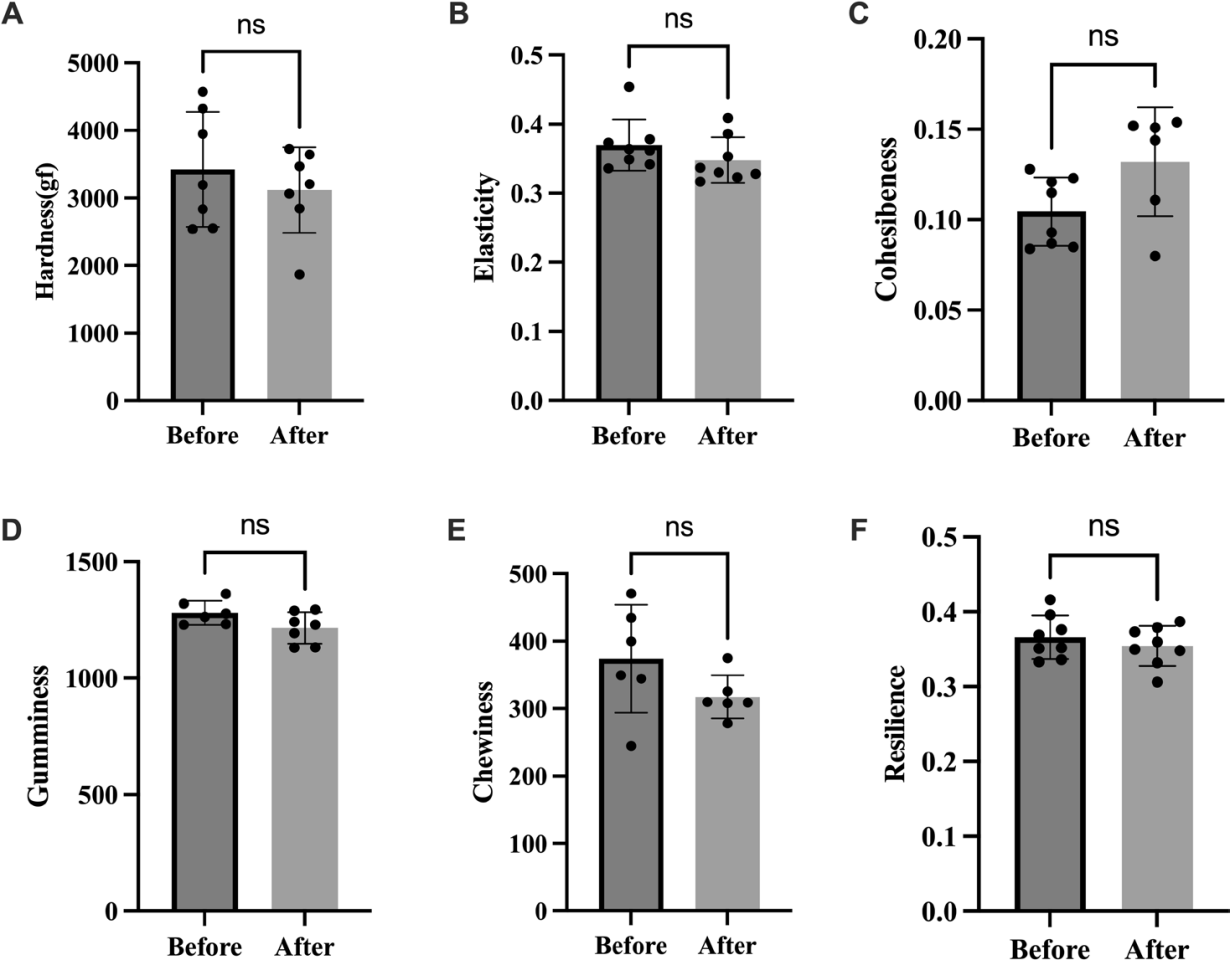
Comparison of changes in sensory indicators of various textures of *X. sorbifolium* kernels before and after debittering. (A) Hardness; (B) elsacticity; (C) cohesiveness; (D) gumminess; (E) chewiness; (F) resilience. Data are expressed as mean ± SD (*n* = 3). The “ns” indicates no significant difference.

**TABLE 5:** Assessment of sensory and physical properties (texture, color, smell, and form) of *X. sorbifolium* kernels before and after the debittering treatment.

Project	Before debittering	After debittering	Difference
Hardness	3490	3082	Slightly reduce
Elasticity	0.375	0.353	Slightly reduce
Cohesiveness	0.396	0.348	Slightly reduce
Gumminess	1295	1211	Slightly reduce
Chewiness	374	318	Slightly reduce
Cohesiveness	0.108	0.139	Slightly increase
Color	Milky white to ivory color	Milky white to ivory color	Basically consistent
Smell	The aroma of roasted almonds and light milk	The aroma of roasted almonds and light milk	Basically consistent
Texture	Initially brittle, becomes soft and tender after chewing	Initially brittle, becomes soft and tender after chewing	Basically Consistent
Form	Aplump and complete shape, with a long diameter of 8–12 mm, and obvious longitudinal stripes on the surface	Aplump and complete shape, with a long diameter of 8–12 mm, and obvious longitudinal stripes on the surface	Basically Consistent

Using a texture analyzer, detailed texture parameters of the nuts were tested and compared, revealing extremely small percentage changes (Figure 6 and Table 5): hardness (decreased by 11.7%), springiness (decreased by 5.9%), cohesiveness (decreased by 12.1%), gumminess (decreased by 6.5%), and chewiness (decreased by 15.0%). These minor fluctuations indicate that the debittering process did not cause significant changes to the core texture characteristics of the nut kernels. It is worth noting that resilience increased by 28.7%, which further helps to enhance the elastic recovery ability. Overall, these results show that the optimized process has excellent compatibility in retaining the inherent texture characteristics, highlighting the advantage of this process in achieving effective debittering without damaging the structural integrity of the nut kernels and the texture attributes related to sensory perception.

## 4 Discussion

The proposed debittering protocol represents a significant advancement over existing methodologies by resolving the longstanding conflict between bitterness elimination and nutrient retention. Under the scope of physical debittering, conventional methods often compromise the bioactive matrix or face application limits. For instance, high‐temperature roasting (150°C–300°C), widely employed for cashews (Kosoko Sup et al. 2014), effectively masks bitterness via the Maillard reaction but risks inducing oxidative rancidity and degrading heat‐sensitive vitamins. Similarly, conduction drying even at moderate temperatures (60°C–80°C) significantly impacts the fatty acid profiles of walnuts (Matin et al. 2024; Ma et al. 2021). Moreover, aqueous leaching techniques—which rely on prolonged passive diffusion—were found by Emojorho (Ernest and Mmuoemene 2022) to cause severe mineral depletion (> 70%) due to non‐selective leakage. In the realm of chemical debittering, conventional solvent extraction leverages non‐polar organic solvents or high‐temperature ethanol to target fat‐soluble bitterants (Cui et al. 2024). Furthermore, pH‐shift extraction effectively isolates protein curds but inevitably alters native protein structures (Sharma et al. 2025). In contrast, our optimized low‐temperature ethanol extraction (40°C) acts as a highly selective, green alternative. It achieves complete debittering with 93.5% trigonelline removal while restricting protein and lipid losses. This precision effectively solvates specific alkaloids without disrupting cellular storage, establishing a new benchmark for the high‐value processing of *X. sorbifolium* kernels.

The key process parameters evaluated in this study exerted predictable effects on trigonelline removal, with plateauing trends observed across different variables, indicating an optimal balance between extraction efficiency and resource utilization. Ethanol concentrations exceeding 40% did not enhance trigonelline removal, which is attributed to the hydrophilic moieties (e.g., amide groups, polar pyridine rings) in trigonelline's molecular structure that form strong hydrogen bonds with water—once a critical ethanol threshold is reached, water alone provides sufficient solvation capacity for trigonelline (Ma et al. 2021; Lu et al. 2022). Similarly, solid–liquid ratios higher than 1:2 failed to improve debittering efficiency, as the process relies on full infiltration of the extraction solution into the kernel matrix; beyond complete submersion of the kernels (Ahmad et al. 2025), increasing solvent volume does not strengthen the mass transfer driving force required for further trigonelline dissolution (Wang and Li 2022).

Temperature and extraction time also exhibited characteristic trends in influencing trigonelline removal. Elevating the temperature from 0°C to 40°C accelerated trigonelline diffusion by enhancing molecular motion, but the removal efficiency plateaued above 40°C due to rate‐limiting factors, including diffusion resistance within kernel tissues and limited matrix infiltration (Fu et al. 2010). In a single‐factor experiment, the extraction equilibrium reaches the optimal extraction time at 16 h. This could be because trigonelline reaches an equilibrium state in the ethanol solvent at this concentration. Prolonging the treatment time offers no extra benefits (Liu et al. 2024). In the orthogonal experiment, at the higher ethanol concentration (60%) needed for this process, the equilibrium time for trigonelline extraction is around 20 h. The final trigonelline concentration is 23.5 mg/kg, and the bitterness score is close to 0. Notably, the method effectively minimized macronutrient degradation: the limited fat loss (3.5%–4.2%) contrasted sharply with the 6%–8% losses observed in thermally processed samples, likely due to the avoidance of high‐temperature‐induced lipid oxidation. Residual bitterness in control groups (bitterness score = 1–2) further highlighted the necessity of precise solvent composition control—for instance, a 60% isopropanol ratio in Control 2 reduced alkaloid solubility, which confirms the solvent polarity‐dependent extraction mechanism reported in previous literature (Bi et al. 2019).

Ethanol's effectiveness as a solvent also addressed the core challenge posed by the high oil content of *X. sorbifolium* kernels, enabling efficient trigonelline extraction while maintaining process feasibility (Zhang et al. 2025). Collectively, these results validate the rationality of the optimized process parameters and the superiority of the proposed method in balancing debittering efficiency, macronutrient preservation, and industrial applicability. Future research should focus on exploring dynamic solvent adjustment strategies to enhance extraction specificity while preserving cost‐effectiveness (Gupta et al. 2023), which will further refine the method and promote its broader application in the food industry.

It should be noted that while the electronic tongue provides objective bitterness quantification, it cannot fully replicate the complex sensory perception of humans. Future research will incorporate trained human sensory panels to validate these findings and assess overall consumer acceptance.

## 5 Conclusion

This study developed a novel debittering method for *X. sorbifolium* kernels, enabling efficient removal of complex bitter compounds while maximizing the retention of nutritional components. *X. sorbifolium* kernels are rich in protein (≈26%), fat (≈56%), and dietary fiber (nearly 18%); however, their application in the food industry is constrained by the presence of bitter substances such as alkaloids and phenolic compounds. Conventional debittering technologies typically result in substantial macronutrient loss and structural damage.

By synergistically combining soaking with optimized solvent extraction, the proposed method achieved complete bitterness elimination (post‐extraction bitterness value = 0), with protein and fat retention rates exceeding 98% and 95%, respectively. Results demonstrated that the optimized treatment conditions (60 vol% ethanol concentration, 20 h extraction time, 40°C extraction temperature) accelerated the release of bitter compounds. Meanwhile, shortened soaking duration and the use of a mixed solvent system effectively minimized macronutrient degradation. The use of food‐grade ethanol as the sole solvent ensures the safety and regulatory compliance of the debittered kernels.

This method features simple operation, suitability for industrial‐scale production, and recyclable solvents, offering favorable economic and environmental benefits. The use of ethanol allows for efficient solvent recovery via simple distillation, reducing operational costs and environmental footprint. Currently, the efficacy and safety of ethanol as an extraction solvent have been well‐established in the processing of edible oils, such as soybean oil (Sawada et al. 2014) and sunflower oil (Baümler et al. 2016), where mature solvent recovery systems are already in place. In contrast to traditional high‐temperature roasting methods, this study employs a mild extraction temperature of 40°C, which effectively minimizes energy consumption while maintaining process efficiency. Future research should focus on optimizing the solvent recovery system to enhance process sustainability. Collectively, these findings provide an effective technical solution for the debittering of *X. sorbifolium* kernels, facilitating their potential application in the food sector.

## Author Contributions

**Xiaowei Zhang:** formal analysis, methodology, investigation. **Shuangyan Zheng:** methodology, supervision. **Hongbing Chen:** data curation, writing – review and editing. **Jinyan Gao:** validation, visualization. **Yi Fang:** methodology, data curation, investigation. **Xin Wang:** methodology. **Yong Wu:** methodology, formal analysis. **Yan Chen:** project administration, methodology, writing – original draft.

## Funding

This work was supported by the Central Funds Guiding the Local Science and Technology Development of Jiangxi Province (20252ZDD020002) and the Research Project of the State Key Laboratory of Food Science and Resources, Nanchang University (Project No. SKLF‐ZZA‐202513). Ganpo Talent Program‐Innovation and Entrepreneurial Leading Talent Project Jiangxi Province (Project No. gpyc20250114).

## Ethics Statement

This study does not involve any human or animal testing.

## Conflicts of Interest

The authors declare no conflicts of interest.

## Data Availability

Data sharing not applicable to this article as no datasets were generated or analysed during the current study.
